# Evidence of COVID-19 Impacts on Occupations During the First Vietnamese National Lockdown

**DOI:** 10.5334/aogh.2976

**Published:** 2020-09-03

**Authors:** Anh Kim Dang, Xuan Thi Thanh Le, Huong Thi Le, Bach Xuan Tran, Toan Thi Thanh Do, Hanh Thi Bich Phan, Thao Thanh Nguyen, Quan Thi Pham, Nhung Thi Kim Ta, Quynh Thi Nguyen, Quan Van Duong, Men Thi Hoang, Hai Quang Pham, Trang Ha Nguyen, Linh Gia Vu, Carl A. Latkin, Cyrus SH Ho, Roger C. M. Ho

**Affiliations:** 1 Institute for Preventive Medicine and Public Health, Hanoi Medical University, Hanoi, VN; 2 Bloomberg School of Public Health, Johns Hopkins University, Baltimore, US; 3 Institute for Global Health Innovations, Duy Tan University, Da Nang, VN; 4 Faculty of Medicine, Duy Tan University, Da Nang, VN; 5 Center of Excellence in Evidence-based Medicine, Nguyen Tat Thanh University, Ho Chi Minh City, VN; 6 Department of Psychological Medicine, National University Hospital, SG; 7 Department of Psychological Medicine, Yong Loo Lin School of Medicine, National University of Singapore, SG; 8 Institute for Health Innovation and Technology (iHealthtech), National University of Singapore, SG

## Abstract

**Background:**

Although “social isolation” protects the life and health of Vietnamese citizens from the adverse effects of the COVID-19 pandemic, it also triggers massive reductions in the economic activities of the country.

**Objective:**

our study aimed to identify negative impacts of COVID-19 on occupations of Vietnamese people during the first national lockdown, including the quality and quantity of jobs as well as adverse problems at work due to COVID-19.

**Methods:**

A cross-sectional study using web-based platforms was conducted during the first time of social isolation in Vietnam at the beginning of April 2020. We utilized a respondent-driven sampling technique to select 1423 respondents from 63 cities and provinces over Vietnam. Exploratory factor analysis (EFA) was used to define sub-domains of perceived impacts of COVID-19 on occupations.

**Findings:**

Approximately two-thirds of respondents reported decreases in their income (61.6%), and 28.2% reported that their income deficit was 40% and above. The percentage of female individuals having decreased revenue due to COVID-19 was higher than that of male respondents (65.2% and 54.7%, respectively). “Worry that colleagues exposed to COVID-19 patients” and “Being alienated because employment-related to COVID-19” accounted for the highest score in each factor. Compared to healthcare workers, being self-employed/unemployed/retired were less likely to suffer from “Increased workload and conflicts due to COVID-19” and “Disclosure and discrimination related to COVID-19 work exposure.”

**Conclusion:**

Our study revealed a drastic reduction in both the quality and quantity of working, as well as the increased fear and stigmatization of exposure to COVID-19 at workplaces. Health protection and economic support are immediate targets that should be focused on when implementing policies and regulations.

## 1 Introduction

Severe Acute Respiratory Syndrome coronavirus 2 (SARS-COV-2 or COVID-19) has detrimental impacts on healthcare systems over the globe and ripple effects on every aspect of human life [1,2]. The COVID-19 pandemic has caused governments to put their countries on an unprecedented pause in at least three months to flatten the contagion curve [3]. Shutdown border, travel restriction, social distancing, and social isolation have been imposed to limit the spread of viruses, which may spark the fear of an impending economic crisis and recession [4,5,6].

Since the number of COVID-19 cases exponentially increased [7], the Government of Vietnam has implemented stringent measures to contain the health crisis, such as encouraging the population to apply strict social distancing measures; school closure at an early stage; suspend all international flights to restrict traveling [8]. On 1 April 2020, the first “social isolation” declared by the Prime Minister was officially implemented, which push all non-essential businesses closing for at least two weeks [9]. Although those containment measures protect the life and health of Vietnamese citizens and may eventually eradicate the COVID-19 from the country, it also causes massive reductions in economic activities. Prospects for the economy and employment are deteriorating rapidly, which is presented in three key domains 1) The number of occupations (unemployment and underemployment); 2) The quality of working (revenues and social protection); and 3) The vulnerable groups experiencing from adverse economic outcomes [10].

Previous studies revealed the devastating impacts of Severe Acute Respiratory Syndrome (SARS) in 2003 on the economy of individual Asian regions such as mainland China, Hong Kong, and Taiwan [11,12]. In addition, other epidemics such as the Ebola virus disease, the Middle East Respiratory Syndrome (MERS), and the rise of infectious pathogens, had catalyzed investments in global health security [13]. The financial losses should be considered as a critical issue during quarantine and social isolation, which may lead to severe socioeconomic distress or trigger symptoms of psychological disorders [14,15]. Moreover, for those who still maintain their occupations, they also have to face the fear of COVID-19 transmission at works or increased workload due to employee reduction [16]. People whose jobs have a high risk of exposure to COVID-19, also experience stigma and discrimination from relatives, family members to the general public [17,18]. Suffering from distress or stigmatization at work may increase the likelihood of developing psychological disorders [17,19].

However, there is a scarcity of evidence regarding the negative impacts of COVID-19 pandemic’s constraints on the jobs of the people. Therefore, our study aimed to address the question of how the COVID-19 has impacted the occupations of Vietnamese people during social isolation, including the quality and quantity of jobs, identifying the vulnerable groups as well as adverse problems at work due to COVID-19. Results from this study will provide critical evidence for policymakers to adapt optimal strategies to mitigate the impacts of COVID-19, even in the resilience stage of the pandemic or further epidemics in the future.

## 2 Materials and Methods

### 2.1 Study settings and participants

At the beginning of April 2020, the Prime Minister of Vietnam imposed the “social isolation” with prompt contact tracing and quarantine, which were considered as the strictest measure ever to eliminating the COVID-19 pandemic. A cross-sectional study was carried out from April 7 to 14, 2020, one week after social distancing and isolation declared in Vietnam. All citizens have been requested to stay at home to curb the virus, only going out when necessary or emergencies, as well as prohibit the gathering of more than two people in public places and mandatory face mask-wearing. Law enforcement agencies were required to strictly enforce social isolation and other rules. In the first stage of combating COVID-19, which was late in January 2020, 16 cases were recorded and hospitalized. In which, 9 cases had epidemiological history related to Wuhan, China, and 7 cases had close contact with 9 cases mentioned above. The lockdown was conducted at the second stage, with a wave of infected people returning from abroad. Respondents who met the following inclusion criteria were selected to take part in the study: 1) Accept the online informed consent to involve in the survey; 2) using a web-based platform to access the online survey; 3) being able to fulfill the questionnaire.

### 2.2 Sample size and sampling method

We used a snowball sampling to select respondents of the study. This technique offered a chain-referral sampling method in which participants recommend other people they know. The initial groups were those currently working (lecturers, administrators, and staff) and students at Hanoi Medical University. A link to the online survey was provided to people of the core groups via messages, social networks, and emails. Core groups had a higher probability of knowing others who had similar socio-economic characteristics and eligible to participate in the survey. After completing the study, they continuously sent the research invitation to their friends and relatives to join in the research via messages, social networks, and emails. The method exploits the social structure to expand the sample size based on tracing the links in the underlying social network. Data put into analysis covered different background information of subjects including health workers, professional educators, white-collar workers, and students over 63 cities and provinces of Vietnam. A total of 1423 respondents took part in the study.

### 2.3 Measure and instruments

#### 2.3.1 Socio-economic characteristics

Our study reflected the socio-economic characteristics of respondents regarding age, gender, marital status, educational level, occupation status, living region, and whether they followed any religion or not.

#### 2.3.2 Perceived impacts of COVID-19 on occupations

Respondents self-reported the impacts of COVID-19 on their income, change in the amount of income, and their occupational status during the epidemic (since at the end of January 2020).

Respondents also answered a range of questions that reflected the effect of the epidemic on their duty at works. Those questions focused on examining their difficulties at work due to COVID-19 (over-workload, stressful, conflict among colleagues), discrimination (avoiding by family and avoiding to share information about employment), as well as the positive attitude about working condition (work spirit, being appreciated by leader/society). Each question was rated from 0 (strongly disagree) to 4 (strongly agree).

### 2.4 Data analysis

Data analysis was carried out with STATA 15.0 (StataCorp LP, College Station, TX). Exploratory factor analysis (EFA) with maximum likelihood estimation was utilized to define interpretable underlying sub-domains of perceived impacts of COVID-19 on occupations. Scree tests, eigenvalues, and differences in model fit were used to explore the number of sub-domains. An orthogonal (varimax) and oblique (geomin) rotation were performed. Cronbach’s alpha described the internal consistency of each factor. There were three sub-domains identified by EFA, including 1) Increased workload and conflicts due to COVID-19 (6 questions); 2) positive attitude towards stability in working condition (4 questions); 3) disclosure and discrimination related to COVID-19 work exposure (4 questions). To compared results between male and female respondents, inferential statistics were applied using the Chi-square test and Mann Whitney test. A Tobit multivariable regression model was used to define factors related to each factor of the EFA. Stepwise forward selection methods were utilized with the cut-off p-value of 0.2. p-value < 0.05 were considered as statistical significance.

### 2.5 Ethical consideration

Informed consent was provided at the beginning of the survey with an adequate introduction of study purpose. If participants agreed to involve, the questionnaire appeared on the next page. By contrast, the survey automatically ended if they refused to participate. The survey was anonymous. All information was kept confidentially and only used for research purposes. The ethic of our study was approved by the Review Committee of the Institute for Preventive Medicine and Public Health, Hanoi Medical University on March 28, 2020.

## 3 Results

Table 1 depicts the socio-economic characteristics of respondents. The majority of respondents lived in the North of Vietnam (79.0%). About one-fourth of respondents were youth (under 25 years old), and the percentage of females was higher than that of males (27.7% and 20.2%, respectively). Undergraduates made up 53.9% of respondents. 24.6% of respondents reported as self-employed/unemployed/retired. The mean age of male respondents was statistically significantly higher than that of female respondents (36.9 [SD = 10.7] and 33.5 [SD = 10.5], respectively).

**Table 1:** Socio-economics characteristics of respondents.

	Male		Female		Total		p value

	n	%	n	%	n	%	

**Total**	203	30.7	459	69.3	662	100.0	
**Region**							
Northern	145	71.4	378	82.4	523	79.0	<0.01
Central	28	13.8	45	9.8	73	11.0	
South	30	14.8	33	7.2	63	9.5	
Foreign	0	0.0	3	0.7	3	0.5	
**Age group**							
Under 25	41	20.2	127	27.7	168	25.4	<0.01
25–34	43	21.2	134	29.2	177	26.7	
35–44	63	31.0	107	23.3	170	25.7	
Above 44	56	27.6	91	19.8	147	22.2	
**Religion**							
Yes	33	16.3	72	15.7	105	15.9	0.85
No	170	83.7	387	84.3	557	84.1	
**Marital status**							
Single	66	32.5	168	36.6	234	35.4	0.25
Living with spouse	133	65.5	274	59.7	407	61.5	
Others	4	2.0	17	3.7	21	3.2	
**Education level**							
High school and below	35	17.2	102	22.2	137	20.7	0.04
Undergraduate	104	51.2	253	55.1	357	53.9	
Postgraduate	64	31.5	104	22.7	168	25.4	
**Occupation**							
Health workers	43	21.2	77	16.8	120	18.1	0.01
Professional educators	38	18.7	108	23.5	146	22.1	
White collar workers	44	21.7	108	23.5	152	23.0	
Students	36	17.7	111	24.2	147	22.2	
Others	42	20.7	55	12.0	97	14.7	
**Occupational status**							
Salaried employee	81	39.9	183	39.9	264	39.9	0.14
Unlimited term full-time contract	48	23.7	79	17.2	127	19.2	
Limited term full-time contract	23	11.3	51	11.1	74	11.2	
Self-employed/Unemployed/Retired	39	19.2	124	27.0	163	24.6	
Others	12	5.9	22	4.8	34	5.1	
	**Mean**	**SD**	**Mean**	**SD**	**Mean**	**SD**	**p value**

**Number of children**	1.3	1.1	1.1	1.0	1.2	1.0	0.04
**Age**	36.9	10.7	33.5	10.5	34.5	10.7	<0.01

Exploratory factor analysis (EFA) models regarding the impacts of COVID-19 on the employment of respondents are presented in Table 2. The Cronbach’s alpha of each sub-domain of increased distress and conflicts due to COVID-19, positive attitude towards stability in working condition, disclosure, and discrimination related to COVID-19 work exposure was 0.76, 0.78, and 0.76, respectively.

**Table 2:** Exploratory factor analysis model of sub-domains regarding impacts of COVID-19 on the employment of respondents.

	Maximum score		Increased distress and conflicts due to COVID-19	Positive attitude towards stability in working condition	Disclosure and discrimination related to COVID-19 work exposure

	n	%			

Enough employees at work to handle all duties	75	11.3		0.72	
Being in good working spirit	74	11.1		0.78	
Being appreciated by the unit leader	32	4.8		0.78	
Being appreciated by the society	27	4.1		0.77	
Worry that colleagues exposed to COVID-19 patient	60	9.0	0.36		
Increase workload	22	3.3	0.80		
Have to work overtime	22	3.3	0.82		
Have to perform duties which never been done before	20	3.0	0.72		
More stressful at work	6	0.9	0.65		
Conflicts occurred among colleagues at work	4	0.6	0.57		
Afraid of sharing with family about risks of exposure to COVID-19 at work	15	2.3			0.54
Being alienated because employment-related to COVID-19	7	1.1			0.85
Relatives being alienated because employment related to COVID-19	7	1.1			0.87
Avoid sharing occupational information	4	0.6			0.74

**Cronbach’s alpha**			0.76	0.78	0.76
**Mean**			2.7	3.4	2.1
**SD**			0.7	0.6	0.7

Table 3 describes the perceived impacts of COVID-19 on employment among respondents. Regarding the effects on income, approximately two-thirds of respondents reported decreases in their income (61.6%). The percentage of females having decreased revenue due to COVID-19 was higher than that of male respondents (65.2% and 54.7%, respectively). Forty-one point seven percent of respondents reported that their income deficit was from 20% to 100%. The mean score of three factors was 2.7 [SD = 0.7], 3.4 [SD = 0.6], and 2.1 [SD = 0.7], respectively. Regarding the factor “Increased distress and conflicts due to COVID-19,” “Worry that colleagues exposed to COVID-19 patients” accounted for the highest score (3.2 [SD = 1.1]). In addition, “Being alienated because employment-related to COVID-19” was rated with the highest score in “Disclosure and discrimination related to COVID-19 work exposure” (2.3 [SD = 1.0]).

**Table 3:** Perceived impacts of COVID-19 on the employment of respondents.

	Male		Female		Total		p-value

	n	%	n	%	n	%	

**Impact of COVID-19 on income**							
Decreased	64	54.7	146	65.2	210	61.6	0.04
Unchanged/Increased	53	45.3	78	34.8	131	38.4	
**Changes in income due to COVID-19**							
Decreased 80–100%	9	7.7	12	5.4	21	6.2	0.12
Decreased 60–80%	11	9.4	12	5.4	23	6.7	
Decreased 40–60%	13	11.1	39	17.4	52	15.3	
Decreased 20–40%	12	10.3	34	15.2	46	13.5	
Decreased <20%	26	22.2	60	26.8	86	25.2	
Unchanged/Increased	46	39.4	67	29.9	113	33.1	
**COVID-19 impact on occupation status**							
Layoffs	8	6.8	21	9.4	29	8.5	0.33
Reduced working hours/shift	38	32.5	65	29.0	103	30.2	
Have to work overtime	14	12.0	16	7.1	30	8.8	
None	57	48.7	122	54.5	179	52.5	
	**Mean**	**SD**	**Mean**	**SD**	**Mean**	**SD**	**p-value**

**Increased distress and conflicts due to COVID-19**	2.7	0.7	2.7	0.6	2.7	0.7	0.04
Worry that colleagues exposed to COVID-19 patients	3.2	1.1	3.2	1.1	3.2	1.1	0.63
Increase workload	2.9	1.0	2.9	0.9	2.9	0.9	0.29
Have to perform duties which never been done before	2.9	1.0	2.7	1.0	2.7	1.0	0.43
Have to work overtime	2.8	1.1	2.5	1.0	2.6	1.0	0.12
More stressful at work	2.5	0.9	2.5	0.9	2.5	0.9	0.76
Conflicts occurred among colleagues at work	2.3	0.9	2.2	0.8	2.2	0.8	0.54
**Positive attitude towards stability in working condition**	3.4	0.7	3.4	0.6	3.4	0.6	0.99
Being in good working spirit	3.6	0.9	3.7	0.7	3.7	0.8	0.72
Enough employees at work to handle all duties	3.6	1.0	3.6	0.9	3.6	0.9	0.20
Be appreciated by the unit leader	3.1	1.0	3.2	0.8	3.2	0.9	0.70
Be appreciated by the society	3.1	0.8	3.2	0.7	3.2	0.8	0.88
**Disclosure and discrimination related to COVID-19 work exposure**	2.2	0.8	2.1	0.7	2.1	0.7	0.06
Being alienated because employment-related to COVID-19	2.3	0.9	2.3	1.0	2.3	1.0	0.63
Afraid of sharing with family about risks of exposure to COVID-19 at work	2.3	1.1	2.0	1.0	2.1	1.0	0.01
Relatives being alienated because employment related to COVID-19	2.1	1.0	2.1	0.9	2.1	0.9	0.68
Avoid sharing occupational information	2.1	0.9	2.0	0.9	2.0	0.9	0.05

Table 4 shows factors associated with the impacts of COVID-19 on employment of respondents. Living with spouse/partners was positively related to a higher score of “Increased distress and conflicts due to COVID-19.” Compared to health workers, professional educators had lower scores on “Increased distress and conflicts due to COVID-19” and “Disclosure and discrimination related to COVID-19 work exposure.” Besides, those who were self-employed/unemployed/retired were less likely to suffer from “Increased workload and conflicts due to COVID-19” and “Disclosure and discrimination related to COVID-19 work exposure.”

**Table 4:** Factors associated with impacts of COVID-19 on employment of respondents.

	Increased distress and conflicts due to COVID-19		Positive attitude towards stability in working condition		Disclosure and discrimination related to COVID-19 work exposure	

	Coef.	95% CI	Coef.	95% CI	Coef.	95% CI

**Gender** (Female vs male)					–0.14*	–0.28; 0.00
**Region** (Central vs Northern)					–0.28**	–0.49; –0.06
**Age group** (vs Under 25)						
25–34			0.12	–0.09; 0.33		
35–44			0.14	–0.12; 0.39		
Above 44			0.25*	–0.01; 0.51		
**Religion** (Yes vs no)	–0.10	–0.24; 0.04				
**Marital status** (Living with spouse vs Single)	0.15**	0.02; 0.29				
**Education level** (High school and below)						
Undergraduate	0.02	–0.16; 0.20				
Postgraduate	0.15	–0.05; 0.35				
**Occupation** (vs Health workers)						
Professional educators	–0.18***	–0.30; –0.05			–0.28***	–0.44; –0.11
White-collar workers			–0.10	–0.23; 0.03		
Students	0.28*	–0.02; 0.59			0.30*	–0.06; 0.66
**Occupational status** (vs Salaried employee)						
Self-employed/Unemployed/Retired	–0.53***	–0.80; –0.26	–0.11	–0.30; 0.09	–0.39**	–0.73; –0.04
Others	–0.17	–0.41; 0.06				
**Number of children**			0.05	–0.03; 0.13		

*** p < 0.01, ** p < 0.05, * p < 0.1.

## 4 Discussion

This study provided critical results of how occupations of Vietnamese citizens have been affected by “social isolation” regulation to mitigate the spread of COVID-19. Yet while the pandemic has subsided and people begin to resume normal life, the economic sequelae still emerge and may persist for a long-term to come. A high percentage of respondents reported a decrease in their revenue, especially among female individuals. A relatively high proportion of respondents were laid off and working hours/shifts cutting with the income loss of 40% and above. Being afraid of colleagues’ exposure to COVID-19 patients was the factor that was mostly increasing distress and conflicts due to COVID-19. For those whose job had a risk of exposure to COVID-19, being alienated because of COVID-19 employment-related problems accounted for the highest score. Additionally, those who were self-employed/unemployed/retired were less likely to suffer from “Increased workload and conflicts due to COVID-19” and “Disclosure and discrimination related to COVID-19 work exposure” compared to healthcare workers.

Economic growth is grinding to a halt, and all countries around the world are undergoing similar experiences, especially in Asia, where the pandemic occurred earliest and later in Europe. In our study, a high proportion of participants reported a reduction in their income during social isolation, in which the income decreased at least 40% in half of them. Our results are similar to a survey conducted in the UK, which revealed that 75% of the self-employed reported having earned less [20]. According to a report of ILO, a preliminary estimate suggested that about 30,000 work months have been lost with the loss of income as a consequence [10]. Overall income losses due to COVID-19 are projected in the range of between 860 and 3,440 billion USD, which affects the continuity of goods and services consumptions of almost all countries [10]. In Vietnam, the widespread decrease in the economy also creates a pervasive negative impact on the working of citizens, with approximately 25.8 million workers are at risk of experiencing a significant reduction in their wages [21]. In addition, a relatively high percentage of respondents underwent job losses and cuts in working hours/shifts in this study. A report of ILO revealed that nearly 1.25 billion employed workers are at an increased risk of drastic layoffs and reductions in working hours, particularly those are in the informal sector with low-paid, low-skilled jobs [22]. Moreover, COVID-19 also has a devastating impact on global unemployment and underemployment. In Vietnam, since the widespread quarantine policy imposed by the government, a range of enterprises have altered their working modalities or closure. These containment measures have triggered a dramatic fall in revenues, working hours, and wages of employees or suspending contracts in non-essential businesses [21]. A survey conducted in 46 provinces and cities of Vietnam also suggested that more than 76% of the participated companies have reduced working shifts or lay-offed their employees [21].

In this study, it is seen that the impacts of COVID-19 on the income of women more devastated than that of men. This is consistent with the previous report, which suggested that females are over-presented in those occupations more affected by COVID-19 (such as food and services), as well as have less access to social protection [10]. Under social isolation and closure of schools or healthcare systems, women will bear a disproportionate burden in the economy due to either cutting wages/layoffs or carrying a different kind of unpaid work at home [23]. Given the predominant roles as caregivers of family, women are often strained with child care, elderly care, and housework; which may contribute to the limit of working hours and economic opportunities [24]. COVID-19 per say does not discriminate, but gender norms and roles may disproportionately shape the amount of disease burden between males and females [25]. Therefore, the ability of women and men to respond to the pandemic, maintain their wellbeing, and economic resilience during the national lockdown will differ.

Moreover, we found that worrying about exposure to COVID-19 patients of colleagues was the factor most strongly associated with increasing distress and conflicts at work due to COVID-19. Our finding is similar to a study in China, which showed that people often generate negative emotions for self-protection from COVID-19 infection [26]. Evidence from the previous epidemic also revealed that stress-related emotions might be stem from over-reaction to public health emergencies [27,28]. Starting with the concerns of their safety, people increase the fear of exposure to viruses from their colleagues at work and are more likely to engage in avoidance behaviors such as limiting communication [29,30].

Those whose job having risks of infecting COVID-19, respondents reported the highest score of being alienated because of their occupational status. Several types of occupations are recognized as high-risk groups to acquire this infection, including health care workers, the staff of the retail, hospitality industry and food services, transport, and security workers [31]. As found in previous outbreaks, participants experienced different types of stigma and discrimination, such as avoiding, treating with fear, suspicion, and critical comments [15,32,33]. Stigma because of exposure to infectious diseases is a significant theme throughout the literature, even after the outbreaks being controlled. People create “parasite avoidance” to prevent contact with those who may carry communicable diseases [34]. In addition, compared to health workers, professional educators were less likely to suffer from “Disclosure and discrimination related to COVID-19 work exposure.” Consistent with previous studies, quarantined health workers tend to report significantly more stigmatization and rejection from people in their local neighborhoods or were unable to resume their job [35], and normal life as families member considered their jobs was too risky [33].

Several implications can be drawn from this study to mitigate the adverse effects of COVID-19 on occupations of people. Health protection and economic support are immediate targets that should be focused on policies and regulations. Policies regarding protecting workers to alleviating the direct effects of the coronavirus should be in line with the WHO recommendations, including the provision of paid leave or wage subsidies to secure income for those who are being quarantined or women who have to take care of children, elderly and other family members. Policymakers should consider gender differences in responding to the COVID-19 pandemic to impose effective, equitable policies and interventions and enhance the empowering of women. Besides, an increase in educational training about the disease and the rationale for quarantine might also be useful to reduce stigmatization, especially at workplaces. Organization needs to prepare workers to return to work after COVID-19 pandemic. Recent study found that a good workplace hygiene and concerns from the organization on physical health status were associated with less psychiatric symptoms in the workforce [36]. Organizations should also invest in wellness programs to reinforce resilience to stigma and other stressors [37].

The strengths of this study were a large sample size and carried out during the first national lockdown in Vietnam. Yet limitations should be considered. High uniformity could occur as the sampling technique relied on re-sharing the research invitation link. Causal interpretation may be limited due to the cross-sectional design. Further studies assessing the change in occupational status and revenue after the “social isolation” should be conducted.

## 5 Conclusion

A high percentage of respondents reported a decrease in their quality and quantity of working, especially among female individuals. Increased fear and stigmatization of exposure to COVID-19 at workplaces were also reported. Health protection and economic support are immediate targets that should be focused on when implementing policies and regulations.
